# Agreement Between an Artificial Intelligence-Based Meal Image Recognition System and the Weighed Dietary Record for Estimating Energy and Nutrient Intakes

**DOI:** 10.3390/nu18060980

**Published:** 2026-03-19

**Authors:** Akiko Sunto, Kiyoharu Aizawa, Yoko Yamakata, Ayaka Iida, Shihoko Suzuki

**Affiliations:** 1 School of Nutrition and Dietetics, Faculty of Health and Social Services, Kanagawa University of Human Services, Yokohama 238-8522, Japan; 2 The University of Tokyo, Tokyo 113-8656, Japan; 3 Information Technology Center, The University of Tokyo, Tokyo 113-8656, Japan

**Keywords:** artificial intelligence, smartphone application, meal image recognition, dietary survey, registered dietitian

## Abstract

**Objectives:** In Japan, smartphone applications are increasingly used for dietary recording in healthcare settings. This study aimed to examine the agreement between energy and nutrient intake estimates obtained using an artificial intelligence (AI)-based dietary recording application and those obtained using the weighed dietary record (WDR). **Methods:** The AI-based dietary recording method (FoodLog Athl method) was compared with WDR. Thirty-six university students (35 women and 1 man) simultaneously recorded their dietary intake using FoodLog Athl (FLA) and the WDR for 10 consecutive days. Energy and nutrient intakes were estimated using each method, and correlations and agreement between the two methods were evaluated. **Results:** Significant positive correlations were observed between the two methods for energy and most nutrients, except for iron, vitamin B1, and sodium chloride equivalent (*p* < 0.01). Compared with the WDR, the FLA method showed systematic overestimation of energy and major macronutrients (protein, fat, and carbohydrate) and underestimation of total dietary fiber. Bland–Altman analysis indicated fixed bias and relatively wide limits of agreement for several nutrients. **Conclusions:** The FLA method demonstrated moderate agreement with the WDR, with systematic bias observed for selected nutrients. These findings suggest that the application may be useful for monitoring overall dietary trends or relative intake over time, but caution is warranted when precise individual-level nutrient quantification is required. Professional review by registered dietitians may help improve estimation accuracy and reduce bias.

## 1. Introduction

Currently, meal recording tools using smartphone applications (apps) are widely utilized by the general public for monitoring the nutritional contents of meals, and several studies have evaluated the ability of these apps to estimate energy and nutrient intakes [1,2,3]. In nutritional management and dietary assessment, the primary concern is not merely the amount of food consumed, but rather the accurate estimation of nutrient intakes, including total energy, macronutrients (carbohydrates, fats, and proteins), and micronutrients.

**Figure 1:**
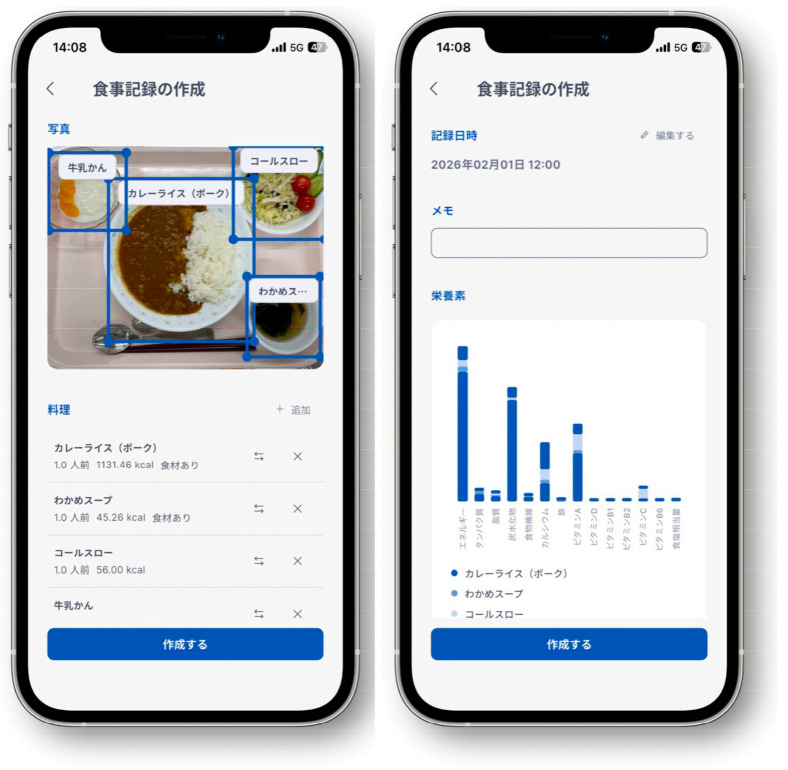
Smartphone screen of the FoodLog Athl meal analysis screen (Japanese interface). The left image shows the recognition of dishes from a meal photograph and their estimated servings and energy. The right image shows the analysis of energy and nutrients, including energy, protein, fat, carbohydrates, total dietary fiber, calcium, iron, vitamins A, D, B1, B2, C, and B6, and salt equivalent. The upper section shows the recording date and time (1 February 2026, 12:00). The screenshot shown reflects the current version of the application interface. During the study period, vitamin B6 was not displayed or included in nutrient estimation. Japanese nutrient labels shown in the interface (エネルギー, タンパク質, 脂質, 炭水化物, 食物繊維, カルシウム, 鉄, ビタミンA, ビタミンD, ビタミンB1, ビタミンB2, ビタミンC, ビタミンB6, 食塩相当量) correspond to energy, protein, fat, carbohydrate, total dietary fiber, calcium, iron, vitamin A, vitamin D, vitamin B1, vitamin B2, vitamin C, vitamin B6, and sodium chloride equivalent, respectively. Japanese dish names shown in the interface (e.g., カレーライス（ポーク）, わかめスープ, コールスロー, 牛乳かん) correspond to pork curry rice, wakame seaweed soup, coleslaw, and milk jelly, respectively.

Some apps can calculate nutritional values instantly using artificial intelligence (AI), with FoodLog Athl (FLA; The University of Tokyo, Tokyo, Japan) being one of them [4]. The FLA is a free app developed and provided to the public by K. Aizawa and Y. Yamakata at the University of Tokyo, and others. It can distinguish areas showing dishes or foods on a meal image and calculate nutritional values in real time. As with FLA, the preceding version, the FoodLog app, also developed by Aizawa et al., was released in 2010 as a meal recording tool that can determine the energy content instantly from a meal’s image [5]. A prior study using FoodLog has shown that a meal recording tool using image recognition technology is easier to use, more user-friendly, and has more frequent viewing of registered meal records than a meal recording tool that has a search function only and does not use image recognition technology. Moreover, this approach is expected to reduce the burden of recording each meal’s nutritional content [6]. In FLA, approximately 140 thousand entries of dishes and foods, including home-cooked meals, meals eaten outside the home, and commercially available products, together with their nutritional values, are registered as a database based on the Standard Tables of Food Composition in Japan 2020 (8th Revised Edition) (Figure 1) [7]. Portion sizes are estimated using a predefined portion-size database integrated into the system, which assigns reference weights to recognized food categories.

**Figure 2:**
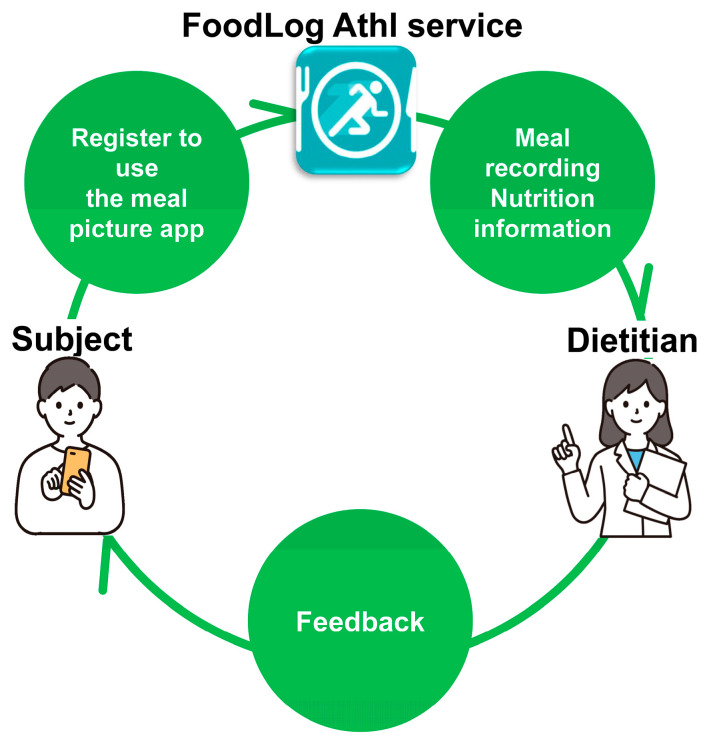
Flowchart of the smartphone app, “FoodLog Athl”.

The AI technology used to link nutritional value data of dishes and foods with meal images recorded by users via FoodLog Athl (FLA) has been adopted by approximately 330 thousand users. In addition to FLA, which is used by individuals to record their meals, another application, FLA for Dietitians, was simultaneously developed as a tool to support nutritional guidance by registered dietitians (Figure 2) [4]. FLA for Dietitians is linked with FLA, allowing dietitians to view users’ meal records in real time once users register the ID of their assigned registered dietitian. Dietitians can also correct meal records when necessary, and users and dietitians can communicate via an in-app chat function. These features suggest that this system may serve as a novel approach to dietary assessment and nutritional guidance.

In contrast, one of the conventional methods used for clinical and research purposes is the weighed dietary record (WDR), in which participants record, in real time, the names and amounts of foods, ingredients, and dishes consumed during a specified period. In addition to the WDR, dietary intake is commonly assessed using methods such as the food frequency questionnaire (FFQ), which records the frequency of consumption of selected foods, and the 24 h dietary recall, in which participants recall and record all foods consumed during the previous 24 h [8].

However, these conventional dietary assessment methods have several limitations, including limited real-time accuracy and a substantial burden on both participants and registered dietitians. The analysis of meal records is time-consuming for dietitians, making it difficult to provide timely feedback. Moreover, because dietary records are often based on self-reported information, underreporting or overreporting frequently occurs [9,10].

AI-based dietary assessment tools are not yet widely used in clinical practice, and the performance of automated estimation of meal quantities remains under investigation. In many cases, the estimation of nutrient intake from food images still relies on manual processing, which is susceptible to human error. Consequently, differences in analytical effort and experience—whether by participants, registered dietitians, or both—can influence the results. Even when participants are able to record meals accurately on their own, registered dietitians may require additional time to develop appropriate dietary improvement plans based on these records. Therefore, AI-based solutions have the potential to enable dietitians to evaluate dietary intake more efficiently, while user-friendly interfaces and simplified procedures may encourage users to continue self-monitoring. Continuous self-monitoring is considered a key factor in the prevention of obesity and diabetes.

However, AI-based dietary assessment systems also have inherent limitations. Image recognition-based dietary assessment relies on multi-step processes—including food detection, segmentation, classification, and portion-size estimation—and errors at each stage may affect intake estimates [11,12]. Previous reviews have also noted variability in performance across different AI models and datasets [13]. These findings highlight the need for continued methodological refinement and careful interpretation of AI-derived dietary estimates. Therefore, AI-based systems may be best positioned as complementary tools rather than direct replacements for traditional dietary assessment methods, particularly when combined with professional review.

If the validity of AI-based meal recording is established and further evidence is accumulated, such approaches may be more widely adopted in nutritional management, reducing the burden on participants and registered dietitians and enabling more timely feedback. Therefore, using the currently released version of FLA, this study aimed to examine agreement in the estimation of energy and nutrient intakes by comparing the AI-based method with the weighed dietary record (WDR).

## 2. Materials and Methods

This study was designed as a pilot agreement study to examine consistency between nutrient intake estimates obtained using the FoodLog Athl (FLA) method and those obtained using the weighed dietary record (WDR).

### 2.1. Participants

Among 38 junior students at the Department of Nutrition, A University, 36 students (35 women and 1 man) who consented to participate in this study and had completed data for both the methods, using FLA and the WDR, were included in this study.

### 2.2. Survey Period

Each participant selected 10 consecutive days from April to July 2023 to perform the meal recording method with FLA and the WDR simultaneously during the 10-day period.

### 2.3. Meal Recording Methods

Before the start of the survey, participants received detailed instructions on how to use FLA and how to perform the WDR, as described below. Using both methods, energy intake and the following nutrients (13 items in total) that were available in the application at the time of data collection were estimated: protein, fat, carbohydrate, total dietary fiber, calcium, iron, retinol activity equivalents, vitamin D, vitamin B1, vitamin B2, vitamin C, and sodium chloride equivalent. For both the FLA method and the WDR, nutrient intake was calculated based on the Standard Tables of Food Composition in Japan (8th edition) [7]. No modifications were made to the original nutrient composition values. The percentage of missing nutrient values was calculated based on the analyzed dataset, defined as the proportion of food-record occurrences (all recorded appearances of food items) for which nutrient values were unavailable.

#### 2.3.1. Recording Method with FoodLog Athl (FLA Method)

During the dietary survey period, participants took photographs of all foods and beverages consumed and uploaded the images to FLA to record their meals. Any items that participants failed to photograph were recorded using the text search function in the app. Participants were instructed to take photographs of their meals prior to consumption only, and they were not required to photograph uneaten food or leftovers. Therefore, the dietary records generated by the FLA system were based solely on pre-consumption images.

The FLA system estimates energy and nutrient intakes by linking recognized food items to a nutrient database based on the Standard Tables of Food Composition in Japan (8th edition) [7]. After food items are identified through image analysis, portion sizes are assigned using a predefined portion-size database integrated into the system, which provides standard reference weights corresponding to each recognized food category. Thus, portion estimation is database-based and does not rely solely on direct volumetric measurement from image pixel data. Nutrient intake is subsequently calculated by linking the assigned portion weight to the corresponding nutrient composition values.

Within the analyzed dataset, the percentage of missing values was 0% for energy, protein, fat, and carbohydrate; 8% for total dietary fiber, calcium, iron, retinol activity equivalents, vitamin D, vitamin B1, vitamin B2, and vitamin C; and 9% for sodium chloride equivalent. Missing values were treated as unavailable and were not imputed. Because the FLA database is continuously updated and the exact configuration of the database at the time of data collection could not be retrospectively reconstructed, the percentage of missing values was calculated based on the analyzed dataset (i.e., all recorded food-item occurrences included in the final analysis).

**Figure 3:**
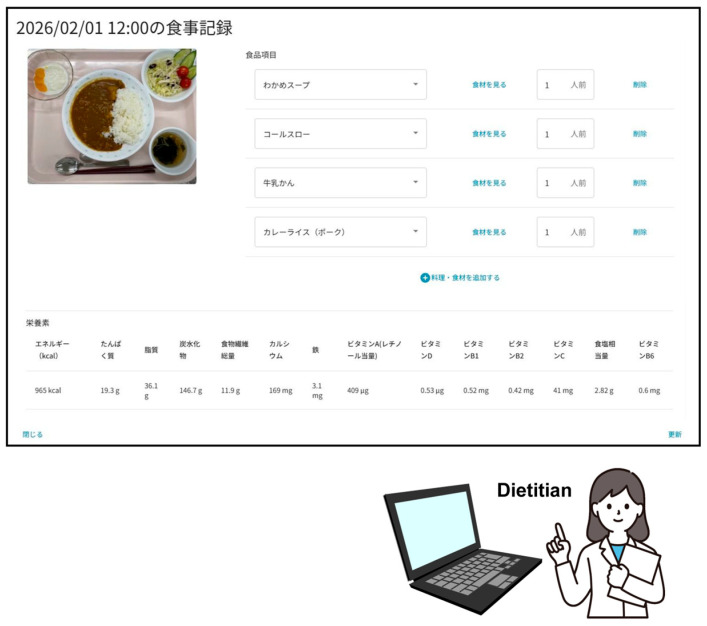
Viewing screen of the meal records on FoodLog Athl for Dietitian (Japanese version). Registered dietitians review and, if necessary, edit the automatically recognized dishes, food items, and their portion sizes. The lower section displays the calculated energy and nutrient values for the meal. The screenshot reflects the current version of the system interface. During the study period, vitamin B6 was neither displayed nor included in the nutrient estimation. Japanese nutrient labels shown in the interface (エネルギー, たんぱく質, 脂質, 炭水化物, 食物繊維総量, カルシウム, 鉄, ビタミンA（レチノール当量）, ビタミンD, ビタミンB1, ビタミンB2, ビタミンC, 食塩相当量, ビタミンB6) correspond to energy, protein, fat, carbohydrate, total dietary fiber, calcium, iron, vitamin A (retinol activity equivalents), vitamin D, vitamin B1, vitamin B2, vitamin C, sodium chloride equivalent, and vitamin B6, respectively. Japanese dish names shown in the interface (e.g., わかめスープ, コールスロー, 牛乳かん, カレーライス（ポーク）) correspond to wakame seaweed soup, coleslaw, milk jelly, and pork curry rice, respectively.

After automatic recognition and portion-size estimation, registered dietitians reviewed participants’ meal records using FLA for Dietitians (Figure 3). Obvious errors in food item selection or portion-size entry were corrected based on professional judgment prior to final nutrient calculation. Corrections were limited to clearly identifiable input errors and did not involve modification of nutrient composition values. The finalized energy and nutrient intake data were then entered into a database for analysis.

#### 2.3.2. Weighed Dietary Record (WDR)

During the dietary survey period, the participants weighed everything that they ingested using a kitchen scale or similar devices, and recorded results on paper. Since the participants were students enrolled in a registered dietitian training program, they calculated intakes of energy and nutrients (13 items in total) based on their training, which would be more efficient than lay users, using the Standard Tables of Food Composition in Japan, and entered them into a database. Subsequently, the calculated values in their database were checked for accuracy by the authors, who were registered dietitians. Participants weighed foods prior to consumption only. Post-consumption weighing was not performed; therefore, leftovers were not directly quantified in the WDR.

### 2.4. Statistical Analysis

Regarding intakes of energy and various nutrients obtained by the two meal recording methods, the FLA method and the WDR, associations between values with the FLA method and those with the WDR were investigated by calculating Spearman’s rank correlation coefficients. To evaluate consistency in intakes of energy and the various nutrients calculated by the FLA and the WDRs, Bland–Altman plots were drawn. Analysis was performed using IBM SPSS Ver. 29 for Windows (IBM Japan, Ltd., Tokyo, Japan) with a significance level of *p* < 0.05.

## 3. Results

Table 1 shows the means and standard deviations of estimated energy and nutrient intakes obtained using the FLA and WDRs, as well as Spearman’s rank correlation coefficients between the two methods. Significant positive correlations were observed between the two methods for energy and most nutrients, except for iron, vitamin B1, and sodium chloride equivalent (*p* < 0.01).

**Figure 4:**
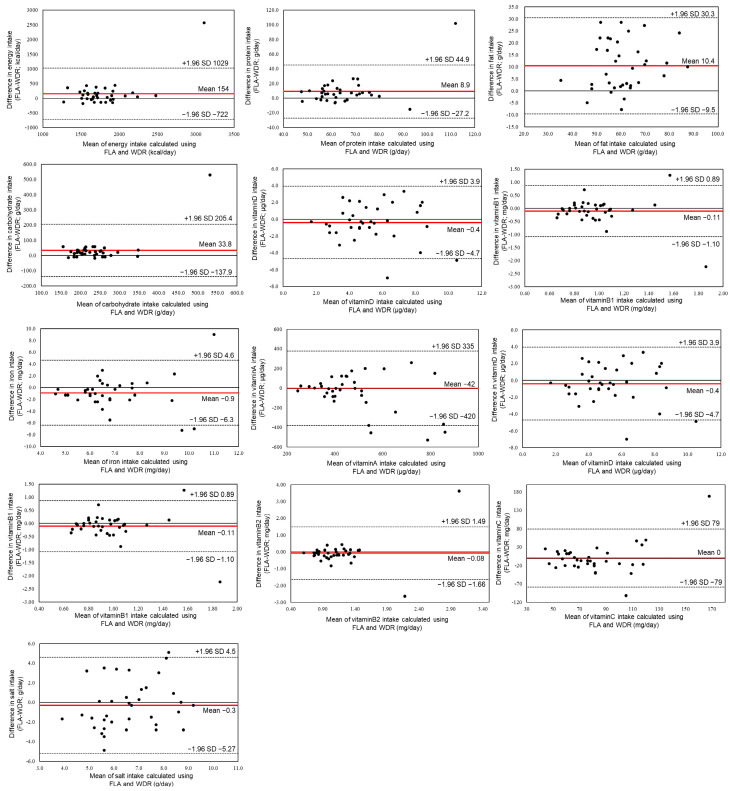
Bland–Altman plots that evaluated consistency between intakes of energy and various nutrients calculated by the FoodLog Athl (FLA) method and those calculated with the weighed dietary record (WDR). The solid lines indicate means, and the dotted lines indicate 95% limits of agreement. Each point represents an individual observation (participant).

Bland–Altman plots used to evaluate agreement between estimated energy and nutrient intakes obtained using the FLA and WDRs are shown in Figure 4. The differences between the two methods (FLA−WDR) are plotted on the vertical axis, and the mean of the two methods is plotted on the horizontal axis.

Positive fixed bias, indicating overestimation by the FLA method, was observed for energy, protein, fat, and carbohydrate. The mean energy intake estimated using the FLA method exceeded that estimated using the WDR by 154 kcal/day (1856 ± 505 vs. 1702 ± 260 kcal/day). Similarly, mean protein, fat, and carbohydrate intakes estimated using the FLA method were higher by 8.9 g/day, 10.4 g/day, and 33.8 g/day, respectively, compared with those estimated using the WDR.

In contrast, a negative fixed bias, indicating underestimation by the FLA method, was observed for total dietary fiber. The mean total dietary fiber intake estimated using the FLA method was 4.5 g/day lower than that estimated using the WDR (13.3 ± 7.0 vs. 17.8 ± 3.6 g/day).

## 4. Discussion

This study found significant positive correlations between energy and most nutrient intake estimates obtained using the FLA method and those obtained using the WDR, except for iron, vitamin B1, and sodium chloride equivalent. These findings indicate that, in this sample of nutrition students, the FLA method demonstrated moderate agreement with the WDR. However, given the pilot design and the convenience sample used in this study, caution is warranted when generalizing these findings to broader populations.

Some foods in the current FLA database do not contain complete information for all nutrients assessed in this study. Micronutrient estimates (e.g., iron and vitamin B1) and sodium chloride equivalent are particularly sensitive to ingredient-level details, seasoning quantities, and cooking methods, which may not be fully captured by image-based assessment. In addition, incomplete micronutrient values in the analyzed dataset and the absence of imputation may have contributed to weaker agreement for these nutrients. Therefore, agreement for selected micronutrients and salt intake should be interpreted with caution, particularly when precise individual-level assessment is required.

Overestimation was also observed for salt intake, which is strongly influenced by individual differences in seasoning practices. Accordingly, accurate estimation of salt intake using AI-based methods remains challenging. These findings suggest that the current version of the FLA method may have limited applicability for precise assessment of certain micronutrients and salt intake without professional review. In clinical contexts such as iron deficiency evaluation or salt reduction programs for hypertension management, AI-estimated values should therefore be interpreted with caution.

More broadly, sodium intake estimation remains challenging in dietary assessment research. Although this study evaluated agreement between the FLA and WDRs, urinary sodium excretion is generally considered a more appropriate reference method for assessing sodium intake. Future validation studies incorporating biomarker-based approaches, such as urinary sodium measurement, would help further clarify the accuracy of sodium estimation using AI-based dietary assessment systems.

The present analysis included energy intake and 12 nutrients that are commonly used indicators in nutritional assessment and meal planning within Japanese institutional foodservice settings, including school lunch programs, and are recognized in the Dietary Reference Intakes for Japanese [9]. These indicators are therefore practically relevant in routine dietary management in Japan.

Although additional nutrients such as omega-3 fatty acids; vitamins B12, A, and E; and minerals including magnesium and zinc were not included in the present agreement analysis, their exclusion does not reflect an inherent technical limitation of the system. Rather, the range of assessed nutrients was determined by the current database configuration and study scope. Future system development and further agreement studies may expand the range of evaluated micronutrients.

Bland–Altman analysis revealed positive fixed bias for energy and the three major macronutrients—protein, fat, and carbohydrate—indicating systematic overestimation by the FLA method. The limits of agreement were relatively wide for several nutrients, reflecting variability at the individual level, and proportional bias was observed for certain macronutrients. These findings indicate that, although the FLA method may be suitable for monitoring relative intake or trends in population-based or behavior-change contexts, caution is required when applying it to precise individual-level dietary counseling in clinical settings.

Previous studies have reported that fat and salt intakes estimated using meal recording applications tend to be overestimated compared with actual intake, particularly in institutional meals such as hospital diets [3]. The weaker agreement observed for fat intake in this study may partly reflect the intrinsic characteristics of lipid-containing foods. Fat content in mixed dishes is strongly influenced by cooking methods and added oils, which are not always visually apparent in meal images. Even small differences in oil quantity can substantially alter estimated fat intake, potentially leading to non-linear discrepancies between methods despite similar portion-size assignments. Consequently, variability in fat estimation may not scale proportionally with total food weight.

Although registered dietitians reviewed the meal records, their role was limited to correcting clearly identifiable input errors, such as obvious food selection mistakes or substantial portion-entry errors. They did not re-estimate portion sizes, reconstruct recipes, or modify nutrient composition values. Thus, professional review functioned primarily as a data quality control measure rather than a recalibration of nutrient estimates. Variability related to cooking methods, oil usage, and database-based portion assumptions may therefore have persisted despite dietitian involvement.

This behavior may have contributed to the differences observed between estimated intakes obtained using the FLA and WDRs. In addition, discrepancies between portion sizes predefined in the FLA database and the actual portion sizes consumed by participants may have influenced the results. Given that nearly all participants were women, actual portion sizes may have been smaller than those predefined in the system, potentially leading to overestimation of energy and macronutrient intakes. Conversely, the negative fixed bias observed for total dietary fiber intake may be related to the selection of foods for which complete nutrient information was unavailable in the FLA database.

Previous studies evaluating image-based dietary assessment applications have reported varying degrees of agreement for energy and macronutrient intake estimation [14,15,16,17]. For example, validation studies of smartphone-based food recording tools such as MyFitnessPal [15] and image-based remote food photography methods (RFPM) [16] have demonstrated moderate agreement with conventional dietary assessment methods. However, direct comparison across applications remains difficult because of differences in study design, database structure, and analytical approaches. The present findings are broadly consistent with this body of literature, suggesting that image-based approaches may provide reasonable estimates of major nutrients under controlled conditions.

In the FLA system, registered dietitians reviewed meal records to correct clearly identifiable input errors but did not re-estimate portion sizes, reconstruct recipes, or modify nutrient composition values. Thus, professional involvement primarily functioned as quality control rather than full recalibration of nutrient estimates. Because initial food recognition and portion-size estimation were performed automatically, the workload associated with professional review was reduced compared with fully manual dietary assessment.

Nevertheless, the scalability of this collaborative approach warrants consideration. As AI models and food databases improve, the extent of required human oversight may decrease. Integration of AI-based estimation with targeted professional review may therefore provide a feasible framework for dietary monitoring in clinical and community settings.

### Limitations

This study has several limitations. First, in this study, post-consumption leftovers were not systematically quantified in either the FLA or WDR. Therefore, discrepancies may have arisen when foods were not fully consumed. In addition, the WDR was performed by participants rather than research staff, which may have introduced measurement error or reporting bias. Although food weighing is widely used in dietary assessment research, it does not represent a biomarker-based reference method. Future studies incorporating objective biomarkers would provide a more rigorous validation framework.

Second, the study sample consisted almost entirely of female students enrolled in a registered dietitian training program (35 women and 1 man). As individuals with formal nutrition education and relatively high health literacy, they may have demonstrated greater compliance and recording accuracy than the general population. Therefore, the agreement observed in this study may not be generalizable to broader populations, including older adults, men, individuals with lower health literacy, and clinical patients. Validation studies in more diverse and representative samples are warranted before broader implementation can be considered.

Third, this study did not include a detailed assessment of user experience, satisfaction, or perceived burden during application use. Future research should evaluate usability and user acceptability to support potential implementation in wider populations.

## 5. Conclusions

This study compared the FLA and WDRs to examine agreement in the estimation of energy and nutrient intakes. Systematic overestimation was observed for energy and the three major macronutrients—protein, fat, and carbohydrate—and moderate agreement was demonstrated overall. These findings suggest that the FLA method may be useful for monitoring relative intake and dietary trends, although caution is warranted when precise individual-level nutrient quantification is required. Incorporating review by registered dietitians may help improve data quality and reduce systematic bias in estimated intakes.

## Figures and Tables

**Table 1:** Average and standard deviation values for each energy and nutrient intake using the FLA method and WDR, and the correlation between the two methods.

Variables	FLA Method	WDR Method	*r*
	Means ± SD	Means ± SD	
Energy (kcal/day)	1856 ± 505	1702 ± 260	0.705 **
Protein (g/day)	69.2 ± 18.9	60.3 ± 10.8	0.545 **
Total fat (g/day)	65.6 ± 12.5	55.3 ± 11.1	0.471 **
Carbohydrate (g/day)	252.5 ± 102.9	218.7 ± 46.3	0.844 **
Total dietary fiber (g/day)	13.3 ± 7.0	17.8 ± 3.6	0.659 **
Calcium (mg/day)	509 ± 510	511 ± 147	0.625 **
Iron (mg/day)	6.4 ± 2.1	7.3 ± 2.0	0.238
Vitamin A ^a^ (µg/day)	461 ± 152	503 ± 218	0.545 **
Vitamin D (µg/day)	5.1 ± 2.2	5.5 ± 2.4	0.592 **
Vitamin B_1_ (mg/day)	0.91 ± 0.31	1.02 ± 0.4	0.113
Vitamin B_2_ (mg/day)	1.10 ± 0.69	1.19 ± 0.47	0.466 **
Vitamin C (mg/day)	80 ± 39	81 ± 26	0.498 **
Sodium chloride equivalent (g/day)	6.4 ± 2.0	6.7 ± 1.6	0.015

FLA, FoodLog Athl. WDR, Weighed Dietary Record. ^a^ Retinol activity equivalent. Spearman’s correlation coefficients (** *p* < 0.01) between the FLA and WDR.

## Data Availability

The data supporting the findings of this study are not publicly available due to ethical and privacy considerations but are available from the corresponding author upon reasonable request.

## Abbreviations

The following abbreviations are used in this manuscript:

App	Application
AI	Artificial intelligence
FLA	FoodLog Athl
WDR	Weighed dietary record
